# Effect of Concomitant Lateral Meniscal Management on ACL Reconstruction Revision Rate and Secondary Meniscal and Cartilaginous Injuries

**DOI:** 10.1177/03635465231194624

**Published:** 2023-09-08

**Authors:** Fabian Persson, Janina Kaarre, Zachary J. Herman, Jonas Olsson Wållgren, Eric Hamrin Senorski, Volker Musahl, Kristian Samuelsson

**Affiliations:** * Department of Orthopaedics, Institute of Clinical Sciences, Sahlgrenska Academy, University of Gothenburg, Gothenburg, Sweden; † Sahlgrenska Sports Medicine Center, Gothenburg, Sweden; ‡ Department of Orthopaedic Surgery, UPMC Freddie Fu Sports Medicine Center, University of Pittsburgh Medical Center, Pittsburgh, Pennsylvania, USA; ‖ Department of Orthopaedics, the NU Hospital Group, Trollhättan, Sweden; ¶ Unit of Physiotherapy, Department of Health and Rehabilitation, Institute of Neuroscience and Physiology, Sahlgrenska Academy, University of Gothenburg, Gothenburg, Sweden; # Department of Orthopaedics, Sahlgrenska University Hospital, Mölndal, Sweden; Investigation performed at Department of Orthopaedics, Institute of Clinical Sciences, Sahlgrenska Academy, University of Gothenburg, Gothenburg, Sweden

**Keywords:** revision ACL reconstruction, meniscal repair, meniscal resection, lateral meniscus

## Abstract

**Background:**

Simultaneous meniscal tears are often present with anterior cruciate ligament (ACL) injuries, and in the acute setting, the lateral meniscus (LM) is more commonly injured than the medial meniscus.

**Purpose:**

To investigate how a concomitant LM injury, repaired, resected, or left in situ during primary ACL reconstruction (ACLR), affects the ACL revision rate and cartilaginous and meniscal status at the time of revision within 2 years after the primary ACLR.

**Study Design:**

Cohort study; Level of evidence, 3.

**Methods:**

Data for 31,705 patients with primary ACLR, extracted from the Swedish National Knee Ligament Registry, were used. The odds of revision ACLR, and cartilaginous as well as meniscal injuries at the time of revision ACLR, were assessed between the unexposed comparison group (isolated ACLR) and the exposed groups of interest (ACLR + LM repair, ACLR + LM resection, ACLR + LM repair + LM resection, or ACLR + LM injury left in situ).

**Results:**

In total, 719 (2.5%) of the included 29,270 patients with 2 years follow-up data underwent revision ACLR within 2 years after the primary ACLR. No significant difference in revision rate was found between the groups. Patients with concomitant LM repair (OR, 3.56; 95% CI, 1.57-8.10; *P* = .0024) or LM resection (OR, 1.76; 95% CI, 1.18-2.62; *P* = .0055) had higher odds of concomitant meniscal injuries (medial or lateral) at the time of revision ACLR than patients undergoing isolated primary ACLR. Additionally, higher odds of concomitant cartilage injuries at the time of revision ACLR were found in patients with LM resection at index ACLR compared with patients undergoing isolated primary ACLR (OR, 1.73; 95% CI, 1.14-2.63; *P* = .010).

**Conclusion:**

The results of this study demonstrated higher odds of meniscal and cartilaginous injuries at the time of revision ACLR within 2 years after primary ACLR + LM resection and higher odds of meniscal injury at the time of revision ACLR within 2 years after primary ACLR + LM repair compared with isolated ACLR. Surgeons should be aware of the possibility of concomitant cartilaginous and meniscal injuries at the time of revision ACLR after index ACLR with concomitant LM injury, regardless of the index treatment type received.

Simultaneous meniscal tears are often present with anterior cruciate ligament (ACL) injuries, and in the acute setting, the lateral meniscus (LM) is more commonly injured than the medial meniscus.^16^ During ACL reconstruction (ACLR), the meniscus may be repaired, resected,^3^ or left in situ based on the type of meniscal injury and patient goals.^25^ Oftentimes, repair is preferred as research has shown improved quality of life scores and decreased rates of osteoarthritis development compared with partial meniscal resection.^18,21^

Unfortunately, failure after primary ACLR is a problem, and patient factors, as well as graft type, femoral notch size, concomitant knee pathology at the time of primary ACLR, and coronal and sagittal malalignment, have been identified as risk factors for ACLR failure requiring revision.^4,12,26^ Furthermore, ACLR failure has also been associated with subsequent damage to menisci and knee cartilage, exacerbating pain and decline in knee function, as well as an increase in the rate of development of posttraumatic osteoarthritis.^8,14,29^ Furthermore, LM deficiency has been associated with persistent knee laxity and increased risk of subsequent injuries.^11^ Although a recent study reported that meniscal pathology at the time of primary ACLR was a risk factor for subsequent cartilaginous damage,^2^ little data exist describing the risk of subsequent cartilaginous and meniscal damage after primary ACLR with LM injury treatment. It is unknown whether differing LM treatment methods in the setting of ACLR affect the cartilaginous and meniscal status in the future. Thus, an increased understanding of the possible association between different LM treatment modalities and subsequent injuries would be beneficial, as surgeons could better understand how differing LM treatment methods impact outcomes in the setting of ACLR.

The purpose of this study was to investigate how concomitant LM injury—repaired, resected, or left in situ during primary ACLR—affects the odds of ACL revision and cartilaginous and meniscal status at the time of revision.

## Methods

This registry-based cohort study was approved by the regional ethical board in Stockholm, Sweden (2011/337-31/3), and the Swedish Ethical Review Authority (2022-00913-01). The study is presented according to the Strengthening the Reporting of Observational Studies in Epidemiology guidelines.^28^

The included data were obtained from the Swedish National Knee Ligament Registry (SNKLR), which primarily aims to collect information on patients undergoing ACLR and includes data on >90% of patients undergoing ACLR in Sweden.^27^ The registry was developed in January 2005 and consists of both surgeon- and patient-reported data, including general medical information, injury information, and surgical characteristics, as well as patient-reported outcome measures. Even though all patient (sex, age, body mass index [BMI]) and injury- and surgery-related data (activity at time of injury, laterality, concomitant injuries, graft type, and fixation type) are reported by the surgeons, the patients are asked to fill out the questionnaires regarding their experience of their current knee function. Information on ACL revision surgery is registered separately and thereafter correlated with that of primary ACLR. Participation in the SNKLR is optional, and exclusion can be requested if a patient does not desire research participation. The registry has been described in more detail in previous literature.^13,17^

### Data Collection and Study Sample

Patients undergoing primary ACLR between 2005 and 2018 with a minimum of 15 years of age at the time of surgery and 2 years of follow-up data were included in this study. However, patients with any previous knee surgery, concomitant fracture, medial meniscal injury, concomitant posterior cruciate ligament, or neurovascular injury were excluded. Also, patients undergoing allograft, synthetic graft, double-bundle ACLR, or surgical treatment for concomitant medial collateral ligament or lateral collateral ligament injury were excluded. The study population was divided into 2 different categories: (1) unexposed comparison group (patients undergoing isolated ACLR without concomitant LM injury), and (2) exposed group (patients undergoing ACLR with concomitant injury to the LM). The exposed group was further divided into 4 different subgroups based on the type of treatment received: (1) meniscal repair (suturing), (2) meniscal resection (meniscectomy), (3) both repair and meniscal resection, and (4) left in situ (nonoperative management). However, no data were available on the specific tear location or number of meniscal sutures used.

Information on patient (age, BMI, and sex), injury (activity at the time of injury), and surgical (graft type and time from injury to surgery) characteristics was additionally obtained from the registry. The activity at the time of injury was further divided into 6 different subcategories: (1) pivoting sport (American football/rugby, basketball, dancing, floorball, gymnastics, handball, ice hockey/bandy, martial arts, racket sports, soccer, volleyball, and wrestling), (2) nonpivoting sport (cross-country skiing, cycling, horseback riding, motocross/endure, skateboarding, snowboarding, and surfing/wakeboarding), (3) alpine/skiing, (4) other physical activity (other recreational sport, exercise, and trampoline), (5) traffic related, and (6) other (outdoor activity and work).

### Outcome Measures

The main outcome of interest was 2-year survival or revision after primary ACLR, where the first occurrence of these 2 options was analyzed. The revision ACLR was defined as an ipsilateral revision ACLR. The secondary outcome of interest was the knee status (concomitant meniscal [medial or lateral] or cartilaginous injuries) at the time of revision ACLR.

### Statistical Analysis

All statistical analyses were performed by using SAS System for Windows software (Version 9.4; SAS Institute). Count and proportion were used to present categorical variables, while mean with standard deviation and median with minimum and maximum were used for presenting continuous and ordinal data, respectively. Univariable logistic regression analysis was used to determine whether concomitant LM injury, repaired, resected, or left in situ during primary ACLR, affects the ACL revision rate and cartilaginous as well as meniscal status at the time of revision ACLR. Results from the univariable logistic regression analyses were presented as odds ratio, 95% CI, and *P* value. The area under the receiver operating characteristic curve (AUC) with 95% CI was further calculated. The AUC varies between 0.5 and 1.00, where a higher number represents a better predictive capacity of the statistical model. For instance, an AUC between 0.5 and 0.7 indicates poor predictive capacity of the statistical model, while an AUC between 0.9 and 1.00 represents excellent predictive capacity. All significance tests were conducted at the 5% significance level.

## Results

### Baseline Characteristics

**Figure 1.**
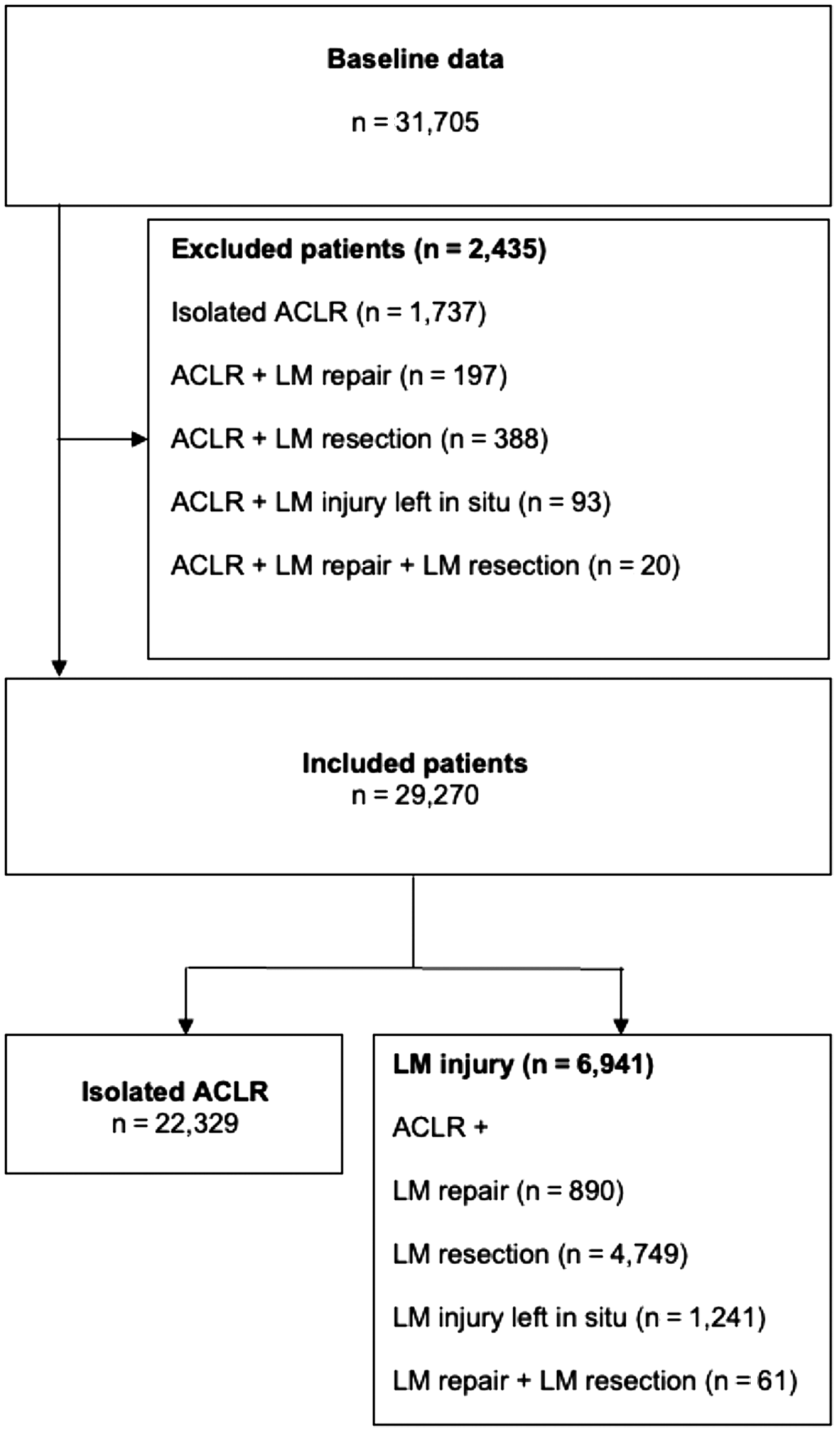
Flowchart. ACLR, anterior cruciate ligament reconstruction; LM, lateral meniscus.

A total of 50,291 patients underwent primary ACLR during this study period. Of these patients, baseline data were available for 31,705 patients, accounting for 63.0% of the total number of patients (Figure 1). Of the included patients (Table 1), 56.1% were male and the mean age at the time of surgery was 27.1 ± 9.9 years. The mean BMI was 24.5 ± 3.3, and the overall time from injury to surgery was 17.0 ± 30.8 months.

**Table 1:** Baseline Characteristics of the Included Patients^*a*^

	Total (N = 31,705)	Isolated ACLR (n = 24,066)	ACLR + LM Repair (n = 1087)	ACLR + LM Resection (n = 5137)	ACLR + LM Injury Left In Situ (n = 1334)	ACLR + LM Repair + LM Resection (n = 81)
Age at time of injury, y	25.6 ± 9.6 23 (1-70)	26.0 ± 9.8 23 (1-70)	22.5 ± 8.3 20 (1-57)	24.7. ± 8.7 22 (7-64)	24.0 ± 8.5 21 (11-58)	21.3 ± 7.3 19 (11-50)
Age at time of surgery, y	27.1 ± 9.9 25 (15-71)	27.6 ± 10.1 25 (15-71)	23.8 ± 8.6 21 (15-58)	26.2 ± 9.1 24 (15-66)	25.2 ± 8.8 23 (15-60)	22.5 ± 7.4 20 (15-50)
Male sex	17,794 (56.1)	12,887 (53.5)	617 (56.8)	3480 (67.7)	751 (56.3)	59 (72.8)
BMI	24.5 ± 3.3 24.2 (15.4-49.8)	24.5 ± 3.3 24.1 (15.4-49.8)	23.8 ± 2.8 23.4 (17.4-35.4)	24.8 ± 3.2 24.4 (16-42.7)	24.4 ± 3.3 23.9 (17.8-44.1)	23.9 ± 3.0 23.4 (19-29.6)
Smoking (yes)^*b*^	784 (5.2)	608 (4.8)	26 (4.3)	117 (4.3)	34 (4.9)	2 (4.3)
Time from injury to surgery, mo	17.0 ± 30.8 7.5 (0-458.4)	17.4 ± 31.3 7.7 (0-458.4)	13.5 ± 27.0 5.9 (0.1-247.9)	16.9 ± 29.7 7.2 (0.1-434.9)	14.7 ± 27.3 7.2 (0.1-358.9)	10.5 ± 19.7 5.4 (0.8-140.8)
ACL graft (yes)						
Patellar tendon autograft	1848 (5.9)	1407 (5.9)	60 (5.6)	310 (6.1)	67 (5.1)	4 (4.9)
Semitendinosus autograft	28,977 (92.4)	22,022 (92.6)	966 (89.9)	4688 (92.2)	1238 (93.6)	63 (77.8)
Quadriceps tendon autograft	525 (1.7)	355 (1.5)	49 (4.6)	89 (1.7)	18 (1.4)	14 (17.3)
Concomitant injury except meniscal injury (yes)	7917 (25.0)	5499 (22.8)	326 (30.0)	1663 (32.4)	386 (28.9)	43 (53.1)
Cartilaginous injury (yes)						
Lateral femoral condyle	1570 (5.0)	837 (3.5)	85 (7.8)	540 (10.5)	94 (7.0)	14 (17.3)
Medial femoral condyle	4218 (13.3)	3083 (12.8)	164 (15.1)	787 (15.3)	162 (12.1)	22 (27.2)
Lateral patella	697 (2.2)	489 (2.0)	32 (2.9)	145 (2.8)	28 (2.1)	3 (3.7)
Medial patella	1276 (4.0)	934 (3.9)	36 (3.3)	223 (4.3)	67 (5.0)	7 (8.6)
Lateral tibial plateau	1754 (5.5)	1026 (4.3)	90 (8.3)	511 (9.9)	112 (8.4)	15 (18.5)
Medial tibial plateau	1058 (3.3)	810 (3.4)	26 (2.4)	193 (3.8)	27 (2.0)	3 (3.7)
Trochlea	743 (2.3)	518 (2.2)	37 (3.4)	156 (3.0)	28 (2.1)	4 (4.9)
Collateral ligament injury (yes)						
LCL	249 (0.8)	159 (0.7)	15 (1.4)	55 (1.1)	19 (1.4)	1 (1.2)
MCL	1189 (3.8)	813 (3.4)	67 (6.2)	212 (4.1)	89 (6.7)	8 (9.9)
PLC injury (yes)	23 (0.1)	16 (0.1)	1 (0.1)	6 (0.1)	00 (0.0)	00 (0.0)
Activity at time of injury (yes)						
Alpine/skiing	4719 (14.9)	3830 (16.0)	156 (14.4)	541 (10.5)	182 (13.7)	10 (12.3)
Pivoting sport^*c*^	21,425 (67.7)	15,881 (66.1)	780 (71.8)	3748 (73.0)	953 (71.5)	63 (77.8)
Nonpivoting sport^*d*^	1301 (4.1)	956 (4.0)	44 (4.1)	234 (4.6)	65 (4.9)	2 (2.5)
Other physical activity^*e*^	1165 (3.7)	915 (3.8)	29 (2.7)	173 (3.4)	46 (3.5)	2 (2.5)
Traffic related	490 (1.5)	364 (1.5)	20 (1.8)	85 (1.7)	19 (1.4)	2 (2.5)
Other^*f*^	2554 (8.1)	2064 (8.6)	57 (5.2)	353 (6.9)	68 (5.1)	2 (2.5)

a Values are given as n (%) for categorical variables and mean ± SD and median (minimum-maximum) for continuous and ordinal variables, respectively. The sums may vary because of missing values. The variables with missing values (n [%] of the total sample) were age at time of injury (712 [2.2]), body mass index (BMI) (15,213 [48.0]), smoking (16,754 [52.8]), time from injury to surgery (751 [2.4]), ACL graft (355 [1.1]), and activity at time of injury (61 [0.2]). ACL, anterior cruciate ligament; ACLR, anterior cruciate ligament reconstruction; LCL, lateral collateral ligament; LM, lateral meniscus; MCL, medial collateral ligament; PLC, posterior lateral corner.

b The numbers are calculated including missing values.

c Pivoting sports included American football/rugby, basketball, dancing, floorball, gymnastics, handball, ice hockey/bandy, martial arts, racket sports, soccer, volleyball, and wrestling.

d Nonpivoting sports included cross-country skiing, cycling, horseback riding, motocross/endure, skateboarding, snowboarding, and surfing/wakeboarding.

e Other physical activity included recreational sports, exercise, and trampoline.

f Other included outdoor activity and work.

### Revision ACLR Rate by Lateral Meniscal Treatment Status at Primary ACLR

Two-year follow-up data were available for 29,270 patients (Figure 1). In total, 719 (2.5%) of the included patients did undergo revision ACLR within 2 years after the primary ACLR. No significant difference in ACL revision rate was found between the patients undergoing ACLR with concomitant LM injury and patients undergoing isolated ACLR (*P* > .05) (Table 2).

**Table 2:** Revision ACLR by Lateral Meniscal Status at Primary ACLR^*a*^

	n (%) of Revisions^*b*^	OR (95% CI)	*P* Value	AUC (95%CI)
Isolated ACLR vs isolated ACLR	530 (2.4)	1.00	.58	
ACLR + LM repair vs isolated ACLR	25 (2.8)	1.19 (0.79-1.79)	.40	
ACLR + LM resection vs isolated ACLR	127 (2.7)	1.13 (0.93-1.38)	.22	
ACLR + LM left in situ vs isolated ACLR	35 (2.8)	1.19 (0.84-1.69)	.32	
ACLR + LM repair + LM resection vs isolated ACLR	2 (3.3)	1.40 (0.34-5.72)	.64	0.51 (0.50-0.53)

a The study group included 29,270 patients. ACLR, anterior cruciate ligament reconstruction; AUC, area under the receiver operating characteristic curve; LM, lateral meniscus.

b Table presents revision within each group, that is, isolated ACLR 530/22,329; ACLR + LM repair 25/890, ACLR + LM resection 127/4,749, ACLR + LM injury left in situ 35/1,241, ACLR + LM repair + LM resection 2/61.

### Concomitant Injury at the Time of Revision ACLR by Lateral Meniscal Treatment Status at Primary ACLR

Data on concomitant meniscal or cartilaginous injuries at revision ACLR in each subgroup of LM treatment at primary ACLR were available for 719 patients. Patients with concomitant LM repair (OR, 3.56; 95% CI, 1.57-8.10; *P* = .0024) or LM resection (OR, 1.76; 95% CI, 1.18-2.62; *P* = .0055) at primary ACLR had higher odds of concomitant meniscal injuries (medial or lateral) at the time of revision ACLR than patients undergoing isolated primary ACLR (Table 3). Additionally, higher odds of concomitant cartilaginous injuries at the time of revision ACLR were found in patients with LM resection compared with patients undergoing isolated primary ACLR (OR, 1.73; 95% CI, 1.14-2.63; *P* = .010).

**Table 3:** Concomitant Meniscal and Cartilaginous Injuries at Revision ACLR by Lateral Meniscal Status at Primary ACLR^*a*^

	n (%) of Meniscal Injuries	OR (95% CI)	*P* Value	AUC (95% CI)
Isolated ACLR vs isolated ACLR	157 (29.6)	1.00	**.0036**	
ACLR + LM repair vs isolated ACLR	15 (60.0)	3.56 (1.57-8.10)	**.0024**	
ACLR + LM resection vs isolated ACLR	54 (42.5)	1.76 (1.18-2.62)	**.0055**	
ACLR + LM left in situ vs isolated ACLR	11 (31.4)	1.09 (0.52-2.28)	.8200	
ACLR + LM repair + LM resection vs isolated ACLR	1 (50.0)	2.38 (0.15-38.22)	.5400	0.56 (0.53-0.60)
	n (%) of Cartilaginous Injuries	OR (95% CI)	*P* Value	AUC (95%CI)
Isolated ACLR vs isolated ACLR	121 (22.8)	1.00	.0680	
ACLR + LM repair vs isolated ACLR	9 (36.0)	1.90 (0.82-4.41)	.1300	
ACLR + LM resection vs isolated ACLR	43 (33.9)	1.73 (1.14-2.63)	**.0100**	
ACLR + LM left in situ vs isolated ACLR	10 (28.6)	1.35 (0.63-2.89)	.4400	
ACLR + LM repair + LM resection vs isolated ACLR	1 (50.0)	3.37 (0.21-54.35)	.3900	0.56 (0.52-0.60)

a The study group included 719 patients. Bold *P* values indicate statistical significance. ACLR, anterior cruciate ligament reconstruction; AUC, area under the receiver operating characteristic curve; LM, lateral meniscus; OR, odds ratio.

## Discussion

The most important finding of this study was that isolated LM injury at the time of ACLR does not appear to increase the risk of revision ACLR at a 2-year follow-up. Additionally, primary ACLR + LM repair was associated with higher odds of subsequent meniscal injuries at the time of revision ACLR compared with isolated ACLR, and primary ACLR + LM resection was associated with higher odds of meniscal and cartilaginous injuries at the time of revision ACLR compared with isolated ACLR.

No difference in ACL revision rates was found between patients undergoing isolated ACLR and patients with combined injury to the ACL and LM. There may be several factors contributing to these study findings. Most importantly, this current study did not include information on the specific type or location of LM injury or the reason for each type of surgery, leading to possible confounding by indication. Also, the current study only aimed to assess the short-term odds of revision, and thus differences in revision rates may have been found at later follow-up time points. However, previous literature is aligned with the current study results, as comparable short-term revision rates between patients undergoing isolated ACLR and patients with concomitant meniscal injuries to those in our study have been reported.^15,19^

In this study, patients with ACLR and concomitant LM resection or LM repair were found to have higher odds of concomitant meniscal injuries (medial or lateral) at the time of revision ACLR than patients undergoing isolated primary ACLR. This could be explained by the possible persistent knee laxity after partial LM resection or the fragility of the repaired meniscus, potentially compromising its structural integrity and reducing its tensile strength, thus increasing its vulnerability to an early injury.^5,20,24^ The crucial role of the LM as a restraint of rotational and dynamic laxity has previously been discussed,^11^ as a significant increase of internal tibial rotation, especially at high degrees of knee flexion, after LM root tears has been reported.^11^ Therefore, our study results may be associated with and explained by the loss of normal function of the LM, leading to greater degrees of knee laxity,^11,20^ resulting in instances where the remaining meniscus is subsequently prone to future injury.

Additionally, higher odds of concomitant cartilaginous injuries at the time of revision ACLR were found in patients with primary ACLR + LM resection compared with patients undergoing isolated primary ACLR. Based on previous literature, the significantly higher risk of early osteoarthritis and chondrolysis in patients treated with ACLR + LM resection, compared with patients with intact meniscus or repaired meniscus, is well known.^6,7,9,10,16,23^ Thus, this could partly explain our study findings suggesting a higher rate of concomitant cartilaginous injuries at the time of revision ACLR, where part of the injuries may be related to loss of normal meniscal function and subsequently increased stress on the articular cartilage. However, despite not having a statistically significant increased chance of cartilaginous injury at the time of revision ACLR, the study cannot conclude that the meniscal repair is chondroprotective. It is also possible that the new ACL injury,^22^ resulting in graft rupture, could be partly responsible for the new cartilaginous damage, and thus the new cartilaginous injury would not be caused only by the previous concomitant LM resection.^1^

The results of this study provide valuable information on the odds of undergoing revision ACLR and future meniscal or cartilaginous damage after ACLR with various treatments of LM injury. Low odds of revision ACLR were found in all the study groups irrespective of treatment of concomitant LM injury. In terms of concomitant meniscal or cartilaginous injuries at the time of revision ACLR within 2 years after primary ACLR, study findings suggest that, when compared with patients undergoing revision ACLR after isolated primary ACLR, those undergoing revision ACLR after primary ACLR + LM resection have a higher tendency to sustain new cartilaginous and meniscal injuries, while those undergoing revision ACLR after primary ACLR + LM repair have higher odds of sustaining new meniscal injuries. Surgeons should be aware of the increased likelihood of persistent knee laxity in the setting of ACLR with concomitant LM injuries and thus should aim to address these injury types, as they are likely to affect the odds of concomitant injuries at the time of revision ACLR. Furthermore, there was no difference in the LM and cartilaginous status between patients with LM injury left in situ and patients undergoing isolated ACLR. It is possible that the tears left in situ were smaller and located in a region of the meniscus that affords an opportunity for healing without intervention. If these tears left in situ successfully heal in the postoperative period, it is likely that these patients would have similar outcomes to those undergoing isolated ACLR. As such, the LM should be preserved as often as possible, by repairing or leaving tears with the potential to heal in situ, to avoid further knee damage in the future.

This study has several strengths and limitations. Most importantly, the study included an overall large sample size including detailed information on patients undergoing ACLR. Furthermore, the SNKLR has previously been reported to include information on >90% of all patients undergoing ACLR in Sweden^27^ and therefore can be considered to represent the Swedish ACLR population. However, the registry nature of the study can be considered a limitation because it limits the possibility of addressing causal relationships. Furthermore, this current study only included information on surgically treated revision cases, leading to a possible underestimation of failure rate and cartilaginous and meniscal damage within 2 years after the primary ACLR. The specific type or location of the LM tear and the reason why some of the meniscal injuries were resected, repaired, or treated nonoperatively were not collected in the registry and thus not included in this current study, leading to possible confounding by indication. Last, specific information on the laterality or severity of meniscal and cartilaginous damage found at the time of revision ACLR was not available.

## Conclusion

The results of this study demonstrated higher odds of meniscal and cartilaginous injuries at the time of revision ACLR within 2 years after primary ACLR + LM resection and higher odds of meniscal injury at the time of revision ACLR within 2 years after primary ACLR + LM repair compared with isolated ACLR. Surgeons should be aware of the possibility of concomitant cartilaginous and meniscal injuries at the time of revision ACLR after index ACLR with concomitant LM injury, regardless of the index treatment type received. Future research is needed to examine the long-term effects of LM injury.
